# REDUCING AVOIDABLE ED VISITS IN OLDER ADULTS—A COLLABORATIVE APPROACH BETWEEN ED AND NURSING HOMES

**DOI:** 10.1093/geroni/igae098.4063

**Published:** 2024-12-31

**Authors:** Hanzhang Xu, Chong Yau Ong, Angus Jun Jie Ng, Gayathri Devi D/O Nadarajan, Marcus Eng Hock Ong, Jean Mui Hua Lee

**Affiliations:** Duke University, Durham, North Carolina, United States; Sengkang General Hospital, Singapore, Singapore; Sengkang General Hospital, Singapore, Singapore; Singapore General Hospital, Singapore, Singapore; Health Services and Systems Research, Singapore, Singapore; Sengkang General Hospital, Singapore, Singapore

## Abstract

Singapore has one of the fastest aging populations in the world. As the number of older adults increases, so does the number of older adults living in nursing homes (NH). Prior research has found that NH residents have a high rate of emergency department (ED) visits and close to half of these visits might be avoidable. To promote high-quality care for NH residents and to address the overcrowding in the ED, we developed a telehealth-based care coordination program named EAGLEcare ACT (Enhancing Advance care planning, Geriatric and end-of-Life care - Acute Care Team). In brief, a public health institution - Sengkang General Hospital has partnered with NHs in their catchment area and delivered telehealth consultations to help NH staff manage acutely-ill NH residents on-site instead of transporting them to the ED. Between Aug 2020 – Dec 2023, we engaged 11 out of the 35 NHs in the catchment area, 7 of which have come on board, and 1166 telehealth consultations were delivered. After the implementation of EAGLEcare ACT, there was a significant decrease (pre: 51/9%, post: 40.5%, P<.001) in the proportion of ED visits from partnering NHs. The impact largely remained throughout the program period. We also found that the effect of EAGLEcare ACT varies by NHs and might be related to differences in the characteristics of NH residents and experiences of NH staff. Findings from this project provided much-needed evidence to enhance care transitions, improve high-quality tele-ED care, and promote health and well-being for older adults residing in NHs.

